# Spontaneous rupture of unscarred uterus in the third trimester after in vitro fertilization-embryo transfer because of bilateral salpingectomy: A case report

**DOI:** 10.1097/MD.0000000000018182

**Published:** 2019-11-27

**Authors:** Yan Sun, Jing Huang, Hong-Fang Kong

**Affiliations:** Department of Obstetrics, Second Hospital of Hebei Medical University, Shijiazhuang, Hebei, People's Republic of China.

**Keywords:** IVF-ET, multiple pregnancy, salpingectomy, spontaneous rupture, unscarred uterus

## Abstract

**Rationale:**

Rupture of an unscarred uterus after in vitro fertilization–embryo transfer (IVF-ET) in a primiparous woman is rare. Assisted reproductive technology (ART)-induced rupture of an unscarred uterus is usually attributable to increased dizygotic twinning rates. Salpingectomy can result in cornual scarring and increase the risk of uterine rupture as well as the mortality rate in a subsequent ectopic pregnancy. Here, we present the first reported case of a spontaneous, third-trimester, uterine rupture in a primiparous woman after IVF-ET due to a history of bilateral salpingectomy because of bilateral oviduct and ovarian cysts; the patient did not have an ectopic pregnancy or any cornual or other uterine scarring during this pregnancy after IVF-ET.

**Patient concerns:**

A 24-year-old woman with a history of IVF-ET and bilateral salpingectomy was admitted to our hospital with unexplained acute upper abdominal pain during the third trimester.

**Diagnosis:**

The fetal heart rate was abnormal. Abdominal ultrasonography was negative. Computed tomography revealed a small amount of abdominal and pericardial effusion. Laboratory tests revealed increased white blood cells. A diagnosis of pregnancy complicated by acute abdomen was considered. Emergent exploratory laparotomy revealed a uterine rupture at the right fundus adjacent to the right cornual area.

**Interventions:**

The patient was successfully managed with simultaneous exploratory laparotomy and lower-segment cesarean section. The rupture site was repaired.

**Outcomes:**

Two live infants were uneventfully delivered. Follow-up assessments of the mother and the female baby on the 42nd postpartum day yielded normal results. The male infant was diagnosed with left hydronephrosis and required an operation.

**Lessons:**

We conclude that the ART-associated increase in dizygotic twinning rates may be a neglected risk factor for spontaneous rupture of the unscarred uterus, especially in patients who have undergone salpingectomy. Uterine rupture should be considered in a patient with multiple pregnancy following IVF-ET who presents with acute abdominal pain and abnormal fetal heart rate. Timely exploratory laparotomy is the key to a good prognosis.

## 1 Introduction

Spontaneous rupture of an unscarred uterus in the third trimester is a rare event.^[1,2]^ The condition has a complex etiology and no typical clinical presentation. Moreover, it is difficult to differentiate from other causes of acute abdomen, which may delay treatment and result in adverse outcomes. However, early diagnosis and prompt treatment are critical, as uterine rupture can be a catastrophic obstetric event, with high fetal and maternal morbidity and mortality.^[3–5]^ Following the development of assisted reproductive technology (ART), its complications are gradually becoming recognized.

Here, we report a case of spontaneous rupture of an unscarred uterus in a primiparous woman who was in the third trimester of her pregnancy and had previously undergone bilateral salpingectomy and in vitro fertilization and embryo transfer (IVF-ET).

## 2 Case presentation

A 24-year-old woman (gravida 2, para 1) who was 34 weeks + 5 days pregnant was admitted to the emergency department of our hospital on 24 July 2018 because of acute upper abdominal pain. The pain had begun the previous day after she had eaten her dinner, and was associated with nausea and vomiting. She had no history of fever. She had had a normal-term delivery 6 years previously. She had undergone bilateral laparoscopic distal salpingectomy because of bilateral oviduct and ovarian cysts 2 years previously. Her menstrual cycle was regular. She had undergone IVF-ET in December 2017 because of tubal-factor infertility caused by bilateral salpingectomy. Two fresh embryos had been transferred. Transvaginal ultrasonography had revealed a dichorionic and diamniotic twin pregnancy with normal cardiac activity and gestational sac situated in a normal uterine cavity. The subsequent course of prenatal care was uneventful. She had no other medical, surgical, or gynecological history.

On admission to our hospital, the patient was in a semi-recumbent position and could not lay down. Her pulse rate was 120 to 140 beats per minute. Her blood pressure varied from 110/70 to 120/80 mm Hg, and her respiratory rate varied from 20 to 25 breaths per minute. Her body temperature was normal. She did not appear pale or feel faint. An abdominal examination revealed mild tenderness in the upper abdomen and a firm abdomen. We did not detect uterine contractions or increased uterine tone. The fetal heart rate was >160 beats per minute. A vaginal examination showed that the cervix was closed, and the fetal membrane was intact. There was no evidence of bleeding or abnormal discharge.

**Figure 1:**
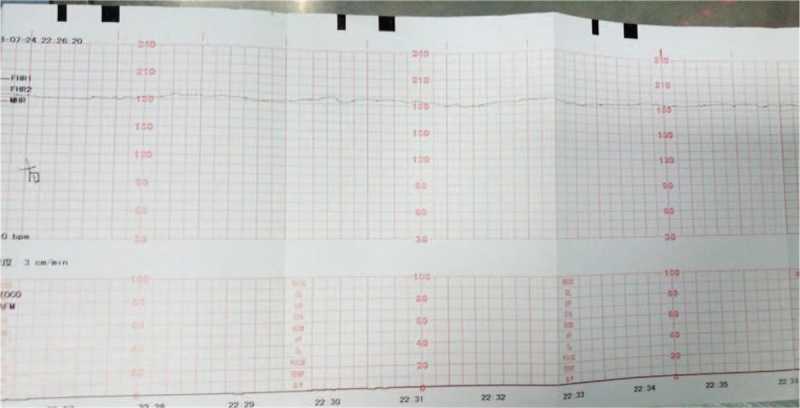
Non-stress test showing persistent increase in heart rate and loss of normal variation in heart rate in the right fetus.

**Figure 2:**
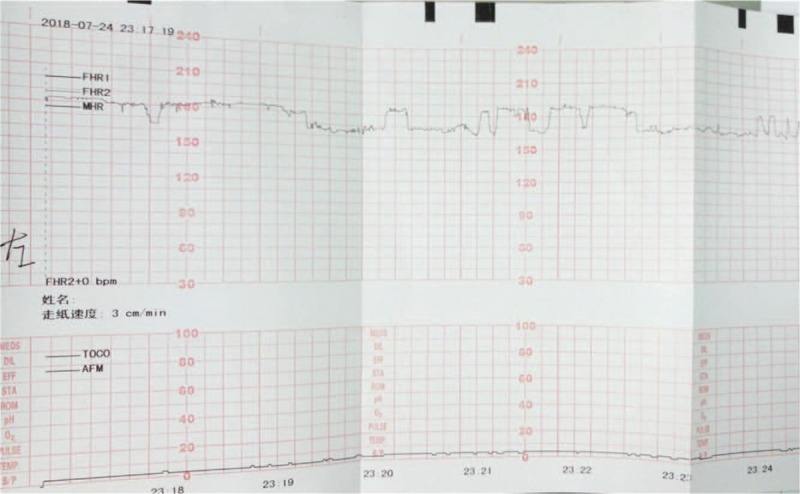
Non-stress test showing persistent increase in heart rate and decrease in normal heart rate variation in the left fetus.

Emergent ultrasonography showed two live fetuses, one in a cephalic presentation and the other in a scapular presentation. The parameters of fetal growth corresponded to 34-week gestation. There were no placental abnormalities. On Doppler examination, vascular flow and amniotic fluid volume were normal. Fetal heart rate ranged from 162 to 178 beats per minute. Laboratory blood tests revealed the following: white blood cells, 16.9 × 10^9^/L; neutrophils, 91.5%; red blood cells, 3.79 × 10^12^/L; hemoglobin, 127.00 g/L; platelets, 272.0 × 10^9^/L; and creatinine, 79 μmol/L. Tests for urine and liver function yielded normal results. There was no evidence of coagulopathy. Abdominal ultrasonography showed fluid (depth, 5.6 cm) surrounding the appendix, and low-dose abdominal computed tomography (CT) revealed a small amount of abdominal effusion. Low-dose chest CT examination showed a small amount of pericardial effusion. As the pain had persisted despite antibiotic and other symptomatic treatments administered by another doctor in the emergency department on the day before admission, a fetal non-stress test (NST) was performed. The NST showed that fetal heart rate in the right fetus was persistently >160 beats per minute and that normal baseline variation of the heartbeat had seriously declined (<5 bpm; Fig. 1). Similar changes were seen in the left fetus (Fig. 2).

**Figure 3:**
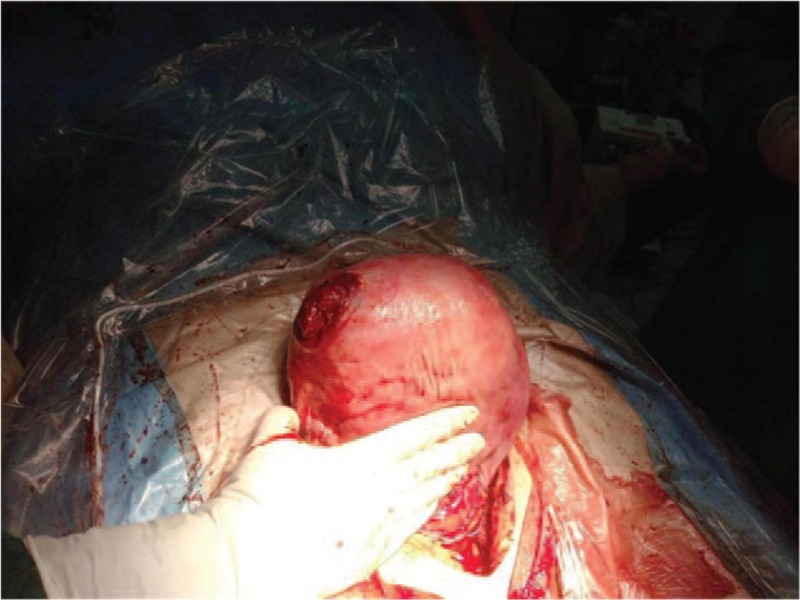
Uterine rupture in the right fundus.

A diagnosis of pregnancy complicated by acute abdomen was considered, and emergent exploratory laparotomy and cesarean section were performed. During surgery, hemoperitoneum (3000 mL) was found in the upper abdominal cavity. After exploration of the intestinal loops, a rupture was palpated on the right side of the uterine fundus, which was covered with blood clots. A lower-segment cesarean section was performed. The fluid of both amniotic sacs was mildly polluted by meconium. Two live infants were uneventfully delivered from the cephalic presentation. The Apgar scores of the older, male infant were 4, 7, and 7 at 1, 5, and 10 minutes. His weight was 2800 grams. The Apgar scores of the female infant were 4, 7, and 8 at 1, 5, and 10 minutes; her weight was 2120 grams. The uterine fundus and cornua were symmetrical, and both placentas were distal to the rupture site. The intact placenta was delivered spontaneously. Uterine examination performed after blood clot removal showed that the 5-cm–wide rupture site was located next to the right cornual area, 7 cm from the proximal end of the right fallopian tube (Fig. 3). The distal ends of the fallopian tubes were absent. The proximal ends of the fallopian tubes, the ovaries, and the left cornu were normal. The rupture site was repaired with two layers of continuous vicryl sutures and 1 layer of continuous vicryl 2–0 sutures. Total intraoperative blood loss was approximately 400 mL. Two units of packed red blood cells, 400 mL fresh frozen plasma, and 6 units of cryoprecipitate were transfused during the operation. There were no further complications.

The postoperative course of the mother was uneventful, and she was discharged on the seventh postoperative day in good condition. The infants required cardiopulmonary resuscitation, intubation, and mechanical ventilation, after which, their vital signs promptly recovered. Both infants were transferred to a tertiary care center and survived. The female infant was discharged from the neonatal intensive care unit on the 14th day of life, without further complications. The male infant was diagnosed with left hydronephrosis and required an operation. He was discharged from the neonatal intensive care unit on the 30th day of life and transferred to the neonatal unit for urinary surgery. Follow-up assessments of the mother and the female baby on the 42nd postpartum day yielded normal results, and an ultrasound examination of the maternal uterus showed normal puerperal changes after cesarean section. The patient had no further complications, but she was advised to avoid future pregnancies because of the high risk of recurrence.

## 3 Discussion

Spontaneous rupture of an unscarred uterus is a rare but noteworthy cause of abdominal pain in women of reproductive age. The global incidence of rupture of an unscarred uterus is 1 in 8000 to 15,000 deliveries, or 0.006%.^[2]^ This incidence is significantly higher in developing countries (e.g., 1 in 131 deliveries in Uganda) than in developed countries (e.g., 1 in 7643–16,849 deliveries in the USA and 12 in 100,000 deliveries in developed countries).^[1–3,6]^

### 3.1 Etiopathogenesis

The pathogenesis and risk factors of unscarred uterine rupture are not well known. The risk factors can be divided in 2 groups: iatrogenic and non-iatrogenic. Iatrogenic risk factors are more common than non-iatrogenic risk factors and include the following^[1–17]^: medical induction or augmentation of labor (e.g., oxytocin stimulation), obstetric maneuvers (e.g., assisted delivery, breech extraction, vacuum extraction), other intrauterine surgeries (e.g., abortion, dilation and curettage, hysteroscopy), myomectomy, adenomyosis, abnormal placentation (e.g., placenta previa, placental abruption, placenta accreta, placenta increta, placenta percreta), dystocia (e.g., obstetric obstruction, prolonged labor), macrosomia, in utero exposure to diethylstilbestrol, chronic corticosteroid use, preterm delivery, delivery after 42 weeks of gestation, and neglected corneal scar after ectopic pregnancy. None of these risk factors was present in our patient.

Our patient had undergone bilateral laparoscopic distal salpingectomy and transfer of 2 fresh embryos because of tubal-factor infertility. However, the uterine rupture site did not involve the cornu but, rather, was located on the right fundus adjacent to the right cornual area. Additionally, the rupture site was located 7 cm from the end of the right fallopian tube. We therefore speculated that cornual scarring from the salpingectomy or a heterotopic pregnancy was not the cause of spontaneous uterine rupture in this patient. We then reviewed the literature on other rare iatrogenic risk factors that might explain the rupture in our patient. We found the following rare iatrogenic factors reported in the literature: multiple gestation^[5,10,11]^ and use of ART^[13,15–17]^ (e.g., ovulation induction, intrauterine insemination, IVF, intracytoplasmic sperm injection). Both these factors were present in our patient. ART has been reported to be associated with an increase in both heterotopic pregnancy and dizygotic twinning rates among patients for whom multiple embryos are transferred and among patients with tubal-factor infertility and a history of salpingectomy.^[13,15–17]^ The incidence of heterotopic pregnancy in patients undergoing ART is 0.2% to 1%;^[16]^ heterotopic pregnancy increases the incidence of salpingectomy and may lead to cornual scarring, thereby increasing the risk of spontaneous uterine rupture in these pregnancies and subsequent pregnancies.^[13,15–17]^ However, our patient had a dichorionic and diamniotic twin pregnancy, with the gestational sacs situated within a normal uterine cavity. Thus, based on the intraoperative findings, cornual scarring and heterotopic pregnancy after IVF-ET were not the cause of spontaneous uterine rupture in our patient. However, ART increases the incidence of multiple gestation,^[5,10,11,13,15–17]^ which is a known risk factor for spontaneous uterine rupture. Hence, we speculated that uterine overexpansion because of multiple pregnancy may have led to uterine rupture in our patient. Although tissue damage due to bilateral salpingectomy was not a direct cause of spontaneous uterine rupture in our patient, it is unclear why a healthy, intact uterus should rupture if there is no damage. Furthermore, we could not totally exclude the possibility of damage caused by the use of an electric cautery during the previous laparoscopic salpingectomy or during the intrauterine manipulation at the time of IVF-ET. Considering all the above factors, we speculated that the rupture of the unscarred uterus in our patient was not attributable to ART-related ectopic pregnancy or cornual scarring after salpingectomy but, rather, to uterine overexpansion caused by multiple pregnancy following IVF-ET and possibly to neglected inadvertent tissue damage in the fundus caused by use of an electric cautery during the previous laparoscopic salpingectomy or during intrauterine manipulation for IVF-ET.

Common non-iatrogenic risk factors for uterine rupture are as follows^[1,2,5–9,12]^: trauma (e.g., domestic violence, traffic accident), uterine anomaly (e.g., bicornuate uterus, uterine septum), cocaine abuse, grand multiparity, and maternal age >35 years. None of these factors was present in our patient.

### 3.2 Clinical manifestation and diagnosis

The clinical presentation of peripartum uterine rupture includes the following manifestations (presented here in order of most to least common): acute abdominal pain and/or abnormal fetal heart rate (most commonly bradycardia), vaginal bleeding, and uterine hypertonus. Less common manifestations include hypotension, hypovolemic shock, hematuria, vomiting, tenderness, and shoulder pain.^[2–5,7,10,12]^ Additionally, signs of uterine rupture may differ, depending on the site of rupture as well as the time since onset. For instance, uterine rupture in the lower segment is associated with the above typical findings (e.g., abnormal fetal heart rate, acute abdominal pain, vaginal bleeding). However, fundal rupture, which leads to the accumulation of blood in the peritoneal cavity, may not be associated with typical clinical findings.^[3,7,10,12]^ As expected, our patient with fundal uterine rupture presented with atypical symptoms including upper abdominal pain after eating, vomiting, nausea, increased pulse rate, and tenderness in the upper abdomen. The fetal heart rate abnormality in our patient prompted us to perform surgery. Although we preoperatively performed ultrasonography and abdominal CT, imaging did not reveal peritoneal hemorrhage. Surgery provided the definitive diagnosis.^[3,7,10,12]^

The differential diagnosis of uterine rupture includes both obstetric and non-obstetric causes. Obstetric causes include bleeding corpus luteum, ectopic pregnancy, molar pregnancy with secondary invasion, placenta percreta, uterine inversion, cervical tear, vaginal tear, coagulopathy, uterine atony, and uterine artery rupture. Non-obstetric causes include other abdominal emergencies such as appendicitis, gallstones, and pancreatitis. Intra-abdominal bleeding is common during the first trimester of an ectopic pregnancy. Hemoperitoneum in the second and third trimester may be attributed to obstetric or non-obstetric causes. The major differential diagnoses in the third trimester and during delivery include placenta previa, placental abruption, uterine atony, uterine inversion, cervical tear, vaginal tear, and other abdominal emergencies.^[4,7,10,12]^

### 3.3 Management and prognosis

Early surgical intervention and effective resuscitation of the mother and fetus are key to the successful treatment of uterine rupture.^[3,12,13]^ The aim of management should be to stop the hemorrhage, repair anatomical damage, and decrease maternal and fetal morbidity and mortality.^[7]^ Depending on the condition of the patient and on clinical circumstances such as the size of the uterine defect, parity, patient age, comorbidities, and expertise of the surgeon, surgical repair of the rupture site or total/subtotal hysterectomy may be performed.^[3,7,12,13]^ Surgical repair is preferred over hysterectomy for patients who hope to have a subsequent pregnancy. However, future pregnancies carry a 4% to 19% risk of recurrence of uterine rupture.^[3,12,13]^ For this reason, it is recommended that women with a history of uterine rupture undergo elective caesarean delivery as soon as fetal lung maturity is reached.^[12]^ Because our patient wished to preserve her reproductive function, we repaired the rupture site and preserved the uterus. The patient was informed about the high risk of recurrence in subsequent pregnancies.

## 4 Conclusion

Spontaneous rupture of an unscarred uterine rupture is an uncommon but potentially life-threatening obstetric event. The clinical presentation is often atypical, but acute abdominal pain and abnormal fetal heart rate are the most common features. IVF-ET, especially in patients with tubal-factor infertility, may be a contributing factor. A diagnosis of uterine rupture should be considered in women who have conceived through IVF-ET and who present with acute abdominal pain and abnormal fetal heart rate. It is difficult to diagnose uterine rupture based on clinical examination alone. Imaging studies such as ultrasonography, CT, and magnetic resonance imaging lack specificity but may facilitate early diagnosis. Once uterine rupture is suspected, surgery should be performed promptly. Exploratory laparotomy and cesarean section with effective maternal and fetal resuscitation may lead to a good prognosis, especially for patients in the third trimester. Surgical repair of the rupture site should be performed for patients who wish to preserve reproductive function. However, depending on the size of the rupture and/or the clinical condition of the patient, hysterectomy may be required. Finally, it may be advisable to rethink the number of embryos that are transferred for patients with infertility, especially for patients with tubal-factor infertility caused by salpingectomy, because multiple pregnancy after salpingectomy increases the incidence of uterine rupture during pregnancy.

## Author contributions

**Resources:** Jing Huang, Hong-fang Kong.

**Supervision:** Yan Sun.

**Writing – original draft:** Yan Sun.

**Writing – review & editing:** Yan Sun.
